# Evaluating the Agreement between Oral, Armpit, and Ear Temperature Readings during Physical Activities in an Outdoor Setting

**DOI:** 10.3390/ijerph21050595

**Published:** 2024-05-04

**Authors:** Yuanzhe Zhao, Leonardo de Almeida e Bueno, David A. Holdsworth, Jeroen H. M. Bergmann

**Affiliations:** 1 Department of Engineering Science, University of Oxford, Parks Road, Oxford OX1 3PJ, UK; yuanzhe.zhao@stx.ox.ac.uk (Y.Z.); leonardo.dealmeidaebueno@eng.ox.ac.uk (L.d.A.e.B.); 2 Oxford University Hospitals NHS Foundation Trust, Oxford OX3 9DU, UK; david.holdsworth@ouh.nhs.uk; 3 Department of Technology and Innovation, TEK, University of Southern Denmark, 5230 Odense, Denmark

**Keywords:** body temperature, oral temperature, tympanic temperature, axillary temperature

## Abstract

Accurate body temperature measurement is essential for monitoring and managing safety during outdoor activities. Physical activities are an essential consideration for public health, with sports taking up an important proportion of these. Athletes’ performances can be directly affected by body temperature fluctuations, with overheating or hypothermia posing serious health risks. Monitoring these temperatures allows coaches and medical staff to make decisions that enhance performance and safety. Traditional methods, like oral, axillary, and tympanic readings, are widely used, but face challenges during intense physical activities in real-world environments. This study evaluated the agreement, correlation, and interchangeability of oral, axillary, and tympanic temperature measurements in outdoor exercise conditions. Systems developed for specific placements might generate different sensor readouts. Conducted as an observational field study, it involved 21 adult participants (11 males and 10 females, average age 25.14 ± 5.80 years) that underwent the Yo-Yo intermittent recovery test protocol on an outdoor court. The main outcomes measured were the agreement and correlation between temperature readings from the three methods, both before and after exercise. The results indicate poor agreement between the measurement sites, with significant deviations observed post-exercise. Although the Spearman correlation coefficients showed consistent temperature changes post-exercise across all methods, the standard deviations in the pairwise comparisons exceeded 0.67 °C. This study concluded that widely used temperature measurement methods are challenging to use during outdoor exercises and should not be considered interchangeable. This variability, especially after exercise, underscores the need for further research using gold standard temperature measurement methods to determine the most suitable site for accurate readings. Care should thus be taken when temperature screening is done at scale using traditional methods, as each measurement site should be considered within its own right.

## 1. Introduction

Being physically active was shown to help prevent and treat a range of noncommunicable diseases (NCDs). The health benefits of sport and physical activity are well established and are often an important part of public health policy [1,2]. At the same time, monitoring has increased during sports to better manage the activities [3]. 

Body temperature is an important indicator of the body’s metabolic state. Deviations from the norm might indicate potential risks, such as heat-related illnesses [4]. Major changes in body temperature can have severe consequences, including exertional heat stroke (a condition that requires immediate medical intervention). The human body’s ability to regulate temperature is a complex interplay of physiological mechanisms, and during intense physical activities, this regulation can be challenging, especially in uncontrolled environments [5,6]. In the demanding world of outdoor sports and physical activities, the accurate measurement and control of body temperature is critical to both performance monitoring and safety management. Recent research studies have increasingly highlighted the critical role it plays in ensuring athlete safety, especially in extreme environments and during adverse weather conditions. For instance, in extreme heat, efficient temperature management, such as pre- or per-cooling [7], can prevent severe conditions, such as heat exhaustion and heat stroke, which are significant risks during intense physical activities [8,9]. Conversely, in cold environments, maintaining an appropriate body temperature is essential to prevent hypothermia and frostbite, which can occur with prolonged exposure [10,11]. 

The accurate monitoring of body temperature is also important for improving sports performance. When athletes compete or train in hot conditions, they experience greater physiological stress than when performing the same activity in milder conditions. In such environments, there is a reduction in cardiac output, central nervous system output, perfusion pressure, and blood flow to exercising muscles, all of which adversely affect exercise performance [12]. An elevated body temperature is a primary factor in inducing exhaustion during exercise in hot conditions. Additionally, beginning exercise with a cooler body temperature can extend the duration an athlete is able to perform before reaching the point of exhaustion [13].

Traditionally, body temperature can be measured through various methods, such as oral, armpit (axillary), tympanic, and rectal readings. Each of these methods presents unique challenges and considerations. 

Although temperatures in sites such as the rectum are thought to be reflective of the core body temperature, these methods are often invasive and inconvenient to measure, especially during exercise [6]. In many cases, especially for leisure time physical activity, equipment for measuring the core body temperature is often lacking. 

Oral temperature is a long-standing, established method for obtaining body temperature, but it is influenced by factors such as recent food or drink intake and breathing patterns and may not always accurately reflect core body temperature during intense exercises [14]. Axillary temperature, while convenient, can be affected by sweat and anatomical variations in soft tissues or blood vessels, and thus, may provide inconsistent results. Whilst tympanic readings, taken in the ear, offer a quick measurement, the reading can be confounded by earwax, an off-axis angle of the thermometer, and environmental conditions [5]. Despite these challenges and site-specific factors, measurements are often taken interchangeably in practice with little consideration of the sites from which they originate. Potential discrepancies may arise in measured temperatures when no information is recorded at the exact site of measurement.

The reliance on these traditional methods during outdoor exercises, particularly under real-world conditions, raises questions about their agreement, correlation, and interchangeability. Outdoor exercises present particular challenges for temperature measurements due to environmental variability. Factors such as ambient temperature, humidity, wind, and exercise intensity can vary widely, influencing the temperature readings across different body locations. These variations may lead to inconsistencies, potential misinterpretations, and challenges in monitoring the physiological response to exercise [15].

The question of whether oral, axillary, and tympanic temperature measurements can be used interchangeably has broad implications for sports performance, safety, and health management. A lack of agreement between different measurement locations may hinder the ability to accurately assess the body condition, especially during intense physical activities. 

In this study, we focused on the agreement and the correlations between oral, armpit, and tympanic readings. By conducting pairwise comparisons between these three positions, both pre-exercise and post-exercise, we aimed to investigate their potential interchangeability.

Our research explored the variations in temperature readings across different body locations, assessing the relationships between locations. We sought to provide insights into the dynamics of temperature changes during outdoor activities, contributing to a more nuanced understanding of temperature monitoring in the context of outdoor sports.

## 2. Methods

### 2.1. Participants

As Table 1 shows, a total of 21 volunteers were recruited for this study; all adults were aged between 18 and 43 with no known health conditions limiting their ability to participate in high-intensity exercise. Informed consent was obtained from all participants prior to the study. All participants wore short-sleeved sportswear on the day of testing.

### 2.2. Settings

Data acquisition was conducted outdoors, with the environmental humidity and temperature recorded before each test. The average ambient temperature across all tests was 20.52 ± 2.03 °C and the average ambient humidity was 81.05% ± 10.61%.

### 2.3. Pre-Test Preparations

Participants were equipped with an oral cavity device for respiratory audio recording and a portable metabolic cart COSMED K5 (COSMED, Rome, Italy).

### 2.4. Body Temperature Measurement

Temperature measurements were taken by the researchers. Readings were obtained before and after a bout of exercise. The following temperatures were measured:

(I) Oral temperature using a digital thermometer (DMT4132, Joytech Healthcare, Hanzhou, China) with an accuracy of ±0.1 °C.
(II) Armpit (axillary) temperature using a digital thermometer (DMT4132, Joytech Healthcare, Hanzhou, China) with an accuracy of ±0.1 °C.
(III) Tympanic (ear) temperature using a tympanic thermometer (Braun Thermoscan 3, Braun, South Boston, MA, USA) with an accuracy of ±0.2 °C.

All the thermometers were used according to the instruction manual provided. For the oral temperature measurement, the probe was placed under the tongue, toward the back of the mouth. The axillary temperature was measured by placing the thermometer probe in the central axillary area of the left arm. To take the ear temperatures with the tympanic thermometer, the probe was gently placed into the left ear canal with gentle forward pressure. Each thermometer was stated to take less than 1 min to establish the temperature. 

### 2.5. Pre-Test Preparations

The initial temperature (temperature before exercise) was measured before the participants began their warm-up. After taking the temperature before exercise, there was a warm-up for subjects, including a familiarization session with the Yo-Yo exercise protocol, which preceded the main exercise test. After the warm-up and familiarization, the main Yo-Yo intermittent recovery test was conducted. 

The Yo-Yo intermittent recovery test was conducted to measure the participants’ ability to perform high-intensity aerobic exercise [16]. The test followed these steps [17]:

1. A 20 m long lane was marked with cones.
2. The test began with a beep from a pre-recorded audio file.
3. Participants ran 20 m back and forth between the cones in time with audible beeps.
4. The running speed was set by the pace of the beeps and increased as the test progressed.
5. Participants were required to complete each 20 m distance before the beep sounded.
6. The test was divided into levels, each lasting around 1–2 min.
7. A short break (10 s) was allowed between each 20 m round trip, during this period participants needed to complete a 10 m walk.

The test continued until participants could no longer complete the 20 m distance before the audible beep and missed the beep twice in a row.

The final temperature (temperature after exercise) was measured on completion of the Yo-Yo test.

### 2.6. Statistical Analysis

The differences in temperature measured by the site-specific thermometers were assessed using a number of recognized error statistics. Mean deviations and limits of agreement were calculated by Bland–Altman analysis. The mean difference (MD) was defined as the average difference between each pair of devices [18,19]:

(1) $$MD=\frac{\sum_{i=1}^{n}\left(T_{dev1,i}-T_{dev2,i}\right)}{n}$$

where i is the index number of a single measurement; n is the total number of the measurements; and $T_{dev1,i}-T_{dev2,i}$ is the difference between recorded temperature between different measurement sites from the i th measurement.

The limit of agreement was calculated by multiplying the standard deviation (SD) of the mean difference between each pair of two groups of devices by 1.96 (which represents the 95% confidence interval). The standard deviation is defined as

(2) $$SD=\sqrt{\frac{\sum_{i=1}^{n}{\left[(T_{dev1,i}-T_{dev2,i})-MD\right]}^{2}}{n-1}}$$

where MD is the mean difference between non-contact tools and reference tools, as calculated in Equation (1).

The difference between the temperature devices were expected to lie within the limits of agreement with a probability of 95%. We also used the Spearman correlation coefficient to test the correlation between groups. All statistical tests were performed using the Python SciPy library 1.10.1.

### 2.7. Ethical Considerations

This study was part of a larger study, which was approved by the Central University Research Ethics Committee (CUREC) (reference: R43470/RE001), and all participants were informed of the study’s purpose, procedures, potential risks, and benefits. Confidentiality was maintained by assigning unique identification numbers to each participant, and all data were stored securely.

## 3. Results

This study was completed by 18 of 21 participants. The temperatures of two participants were not measured. And one participant did not complete the full test. Descriptive statistics for temperature readings are presented in Table 2.

The pairwise comparisons between the oral, armpit, and tympanic temperature readings were conducted to evaluate their agreement between pre-exercise and post-exercise. Bland–Altman analysis plots provide a visual representation of the agreement between the methods by plotting the differences against the averages of the two methods. The limits of agreement, which are the mean difference ±1.96 times the SD, were also calculated and are shown on the plots. Scatter plots were generated to visually expose any monotonic relationships between the different temperature measurement sites. The findings are detailed below.

### 3.1. Comparison of Oral and Armpit Temperature Readings

**Figure 1:**
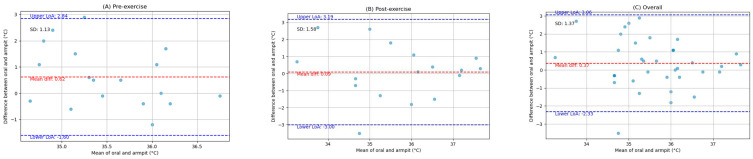
Bland-Altman plots indicating the mean difference (red dashed line) and limits of agreement (blue dashed lines) for the comparison between oral and armpit measurements: (**A**) pre-exercise, (**B**) post-exercise, and (**C**) overall.

**Figure 2:**
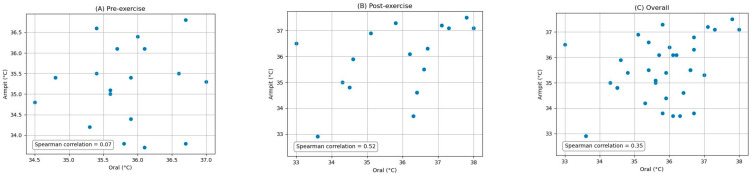
Scatter plots of the comparison between oral and armpit measurements: (**A**) pre-exercise, (**B**) post-exercise, and (**C**) overall.

**Figure 3:**
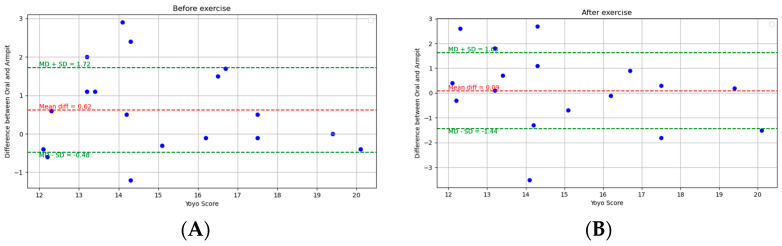
Scatter plots of the Yo-Yo test score and temperature difference between oral and armpit readings: (**A**) pre-exercise and (**B**) post-exercise.

Figure 1 depicts the Bland–Altman analysis of the comparison between the oral and armpit readings of two different states: pre-exercise and post-exercise, as well as the overall comparison between these sites. And Figure 2 shows the scatter plots of the comparison between these locations in corresponding periods. Figure 3 shows the scatter plots of the Yo-Yo test score against the temperature difference between the oral and armpit readings before and after exercise.

**Overall:** The mean difference (MD) between the oral and armpit temperature readings across all measurements was 0.37 °C, with an associated standard deviation (SD) of 1.37 °C. The overall Spearman correlation coefficient was 0.35 (*p*-value = 0.04).

**Pre-exercise:** Before exercise, the MD between the two methods was 0.62 °C, with an SD of 1.13 °C. The Spearman correlation coefficient was 0.07 (*p*-value = 0.77).

**Post-exercise:** After exercise, the MD reduced to 0.09 °C, with an SD of 1.58 °C. The Spearman correlation coefficient was 0.52 (*p*-value = 0.03).

### 3.2. Comparison of Oral and Tympanic Temperature Readings

**Figure 4:**
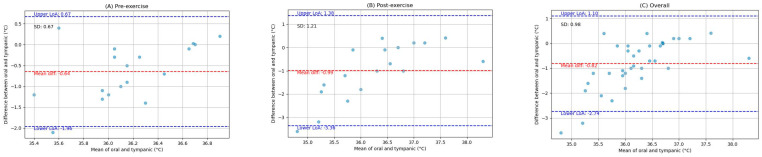
Bland-Altman plots indicating the mean difference (red dashed line) and limits of agreement (blue dashed lines) for the comparison between oral and tympanic measurements: (**A**) pre-exercise, (**B**) post-exercise, and (**C**) overall.

**Figure 5:**
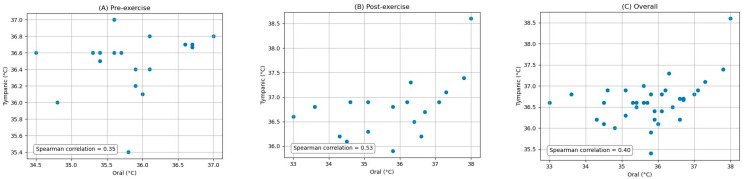
Scatter plots of the comparison between oral and tympanic measurements: (**A**) pre-exercise, (**B**) post-exercise, and (**C**) overall.

**Figure 6:**
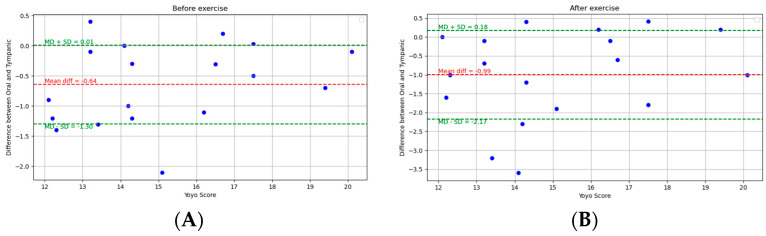
Scatter plots of the Yo-Yo test score and temperature difference between oral and tympanic readings: (**A**) pre-exercise and (**B**) post-exercise.

Figure 4 depicts the Bland–Altman analysis of the comparison between the oral and tympanic readings of two different states: pre-exercise and post-exercise, as well as the overall comparison between these sites. And Figure 5 shows the scatter plots of the comparison between these locations in corresponding periods. Figure 6 shows the scatter plots of the Yo-Yo test score against the temperature difference between the oral and tympanic readings before and after exercise.

**Overall:** For the oral and tympanic methods, the overall MD was −0.82 °C, with an SD of 0.98 °C. The Spearman correlation coefficient was 0.40 (*p*-value = 0.02).

**Pre-exercise:** Before exercise, the MD was −0.64 °C, with an SD of 0.67 °C. The Spearman correlation coefficient was 0.35 (*p*-value = 0.15).

**Post-exercise:** After exercise, the MD increased to −0.99 °C, with an SD of 1.21 °C. The Spearman correlation coefficient was 0.53 (*p*-value = 0.02).

### 3.3. Comparison of Armpit and Tympanic Temperature Readings

**Figure 7:**
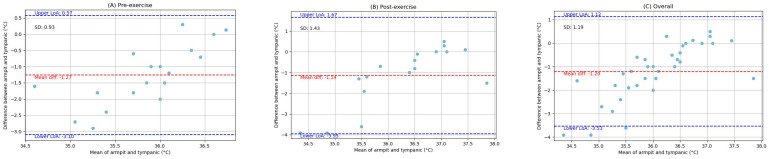
Bland-Altman plots indicating the mean difference (red dashed line) and limits of agreement (blue dashed lines) for the comparison between armpit and tympanic measurements: (**A**) pre-exercise, (**B**) post-exercise, and (**C**) overall.

**Figure 8:**
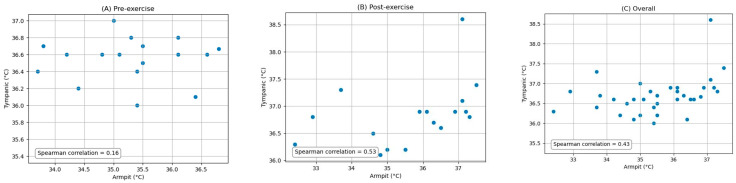
Scatter plots of the comparison between armpit and tympanic measurements: (**A**) pre-exercise, (**B**) post-exercise, and (**C**) overall.

**Figure 9:**
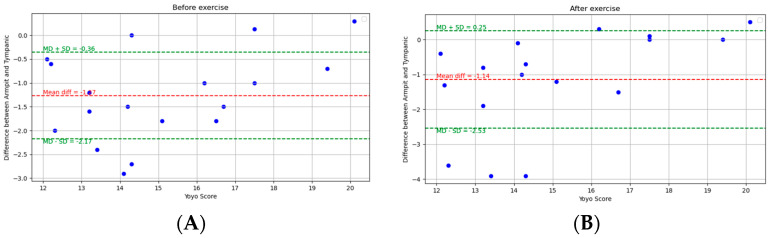
Scatter plots of the Yo-Yo test score and temperature difference between armpit and tympanic readings: (**A**) pre-exercise and (**B**) post-exercise.

Figure 7 depicts the Bland–Altman analysis of the comparison between the oral and tympanic readings of two different states: pre-exercise and post-exercise, as well as the overall comparison between these sites. And Figure 8 shows the scatter plots of the comparison between these locations in corresponding periods. Figure 9 shows the scatter plots of the Yo-Yo test score and temperature difference between the armpit and tympanic readings before and after exercise.

**Overall:** When comparing the armpit and tympanic readings, the overall MD was −1.20 °C, with an SD of 1.19 °C. The Spearman correlation coefficient was 0.43 (*p*-value = 0.01).

**Pre-exercise:** Before exercise, the MD was −1.27 °C, with an SD of 0.93 °C. The Spearman correlation coefficient was 0.16 (*p*-value = 0.52).

**Post-exercise:** Post-exercise measurements showed an MD of −1.14 °C and an SD of 1.43 °C. The Spearman correlation coefficient was 0.53 (*p*-value = 0.03).

## 4. Discussion

The accurate measurement of body temperature during outdoor exercises, especially under conditions that are uncontrolled (i.e., typical real-world scenarios), is of paramount importance. This importance is not only rooted in the ability to monitor performance but also in ensuring the welfare of those that are completing demanding tasks. Our study directly focused on the agreement and correlation between oral, armpit, and tympanic temperature measurements.

Our results indicate that the oral and armpit temperatures were generally lower than the tympanic temperatures, both before and after exercise, which confirmed the conclusion of a previous study in this domain [20]. However, the differences between the oral and armpit temperatures did not reach statistical significance, complicating any definitive assertions about which of these two sites consistently recorded higher or lower temperatures. The lack of a significant difference might have been influenced by several factors, including the measurement technique, the environmental conditions, and the individual anatomical and physiological characteristics of the participants.

We found that there was a marked difference in terms of the agreement between the three most commonly used temperature measurement methods. The limits of agreement (LoAs), calculated as the mean difference ± 1.96 times the standard deviations (SDs), offered a quantitative measure of this agreement and showed that the values lay well outside the clinically acceptable standard, where the limits of agreement are normally considered to be less than ±0.5 °C [21]. Scatter plots further demonstrated the differences between the temperature measurements at different sites, indicating that each site had its own “unique” local temperature environment.

In clinical lab settings, the temperature readings from different locations typically differ by no more than ±0.3 °C [22]. However, our results showed a large deviation across measurement locations. The mean difference across all comparisons exceeded ±0.67 °C. The manuals from the manufacturers of these devices claimed an accuracy within ±0.1 °C in the temperature range 18 °C to 28 °C. This implies that the factors in an outdoor exercise setting introduce variations that substantially reduce the accuracy of temperature measurement in the oral cavity, axilla, and middle ear. Local differences for each measurement site may explain the poor between-site agreement that was found in this study. 

A salient observation from our results was the consistent increase in variability in the pairwise comparisons of post-exercise temperature readings across the three measurement sites compared with their pre-exercise measurements. This was evidenced by the larger SD post-exercise. Such a pattern suggests that the physiological responses to exercise, potentially combined with external environmental factors, differently affected each measurement location. Factors like increased blood flow, metabolic rate changes, and sweating can influence temperature readings across various body locations in different ways [23,24].

The strength of the Spearman correlations between the different temperature sites was consistently lower before exercise compared with post-exercise. This was true across all comparisons, suggesting a stronger monotonic relationship between the sites post-exercise. This could imply that the relative changes in temperature readings across different methods might follow a more “similar” trajectory after physical exertion, despite the inherent variability in the obtained absolute readings. A possible explanation for this is that the hyperthermic stimulus of exercise provides a more powerful signal to determine the absolute temperature at all body sites, reducing the effect of inter-individual variation at rest and increasing the signal-to-noise ratio.

Given the observed between-site discrepancies observed in this study, it would be prudent to treat any approach that uses temperature measurements at interchangeable sites with caution. The poor agreement in temperature at different locations demonstrates the importance of treating these different methods as providing discrete temperature parameters. There may be scenarios in which these methods offer similar insights. However, their inherent variability, especially post-exercise, suggests that they might not always be used interchangeably without careful consideration. 

Although physical exertion increased the difference between the different measurement sites, our results show that these variations did not directly relate to changes in physical performance, as assessed by the Yo-Yo test. This could indicate that other physiological factors or environmental conditions may play a more dominant role in influencing the Yo-Yo test outcomes. Additionally, it is possible that the Yo-Yo test, which primarily evaluates an individual’s endurance capacity, may not be sensitive enough to reflect subtle effects that temperature differences could have on performance metrics [16]. 

A significant limitation of our study was the absence of a gold reference standard, such as esophageal, rectal, or ingestible gastrointestinal thermometry. These are often considered as the best core body temperature references that are obtainable in a clinical setting [25,26]. However, these methods are also more invasive and challenging to measure [27]. They are also not often applied in daily data collection during exercise, and therefore, are not easy to apply in real-world situations. More importantly, this study was concerned with devices that were specifically developed for a given (or multiple) site(s) and to what extent these systems provided an interchangeable method. Several studies suggested a poor agreement between non-invasive body temperature measurement and core temperature during exercise [5,6]. Our reliance on commercially available thermometers should make our findings more generalizable to real-world scenarios and allows us to reflect on the system engineering principle of external validation. However, it might also introduce more uncertainty with regard to the actual core temperature, as no gold reference measurement was used in our study. New and accurate devices for measuring core body temperature have started to emerge. These include the CALERA Research body temperature sensor and the CORE body temperature sensor developed by greenTEG AG, Rümlang, Switzerland, which are based on the calculation of heat flux [28,29]. These innovative devices offer non-invasive, continuous monitoring of core body temperature during exercise. However, further research is necessary to validate their performance with comparison to traditional core body temperature measurement methods to ensure reliability.

Furthermore, while it is not possible to regulate outdoor ambient temperature and humidity, these elements play a crucial role. Changes in ambient temperature affect the difference between the body surface and core body temperature readings, with lower ambient temperatures amplifying this difference [30,31]. In our study, the ambient temperature during our tests averaged 20.52 ± 2.03 °C, which is typically cooler than summer temperatures in many countries [32]. Thus, our findings should be interpreted with caution in these contexts, and larger studies should be conducted to obtain a more complete picture. 

Environmental conditions aside, factors like age and gender also influence body temperature measurement accuracy, necessitating further research for a broader set of participants. For instance, older individuals tend to have increased cutaneous blood flow at rest compared with their younger counterparts. This causes higher skin temperatures, largely due to decreased cutaneous sympathetic nerve activity [33]. Additionally, women typically have cooler skin temperatures than men, which is a phenomenon linked to their higher body fat percentages [34].

There is a strong need to promote health and well-being by encouraging communities to become more active. At the same time, with more extreme weather conditions, more focus is needed on the appropriate monitoring of the population during these activities. Accurate data can help with providing better guidance with regard to being active under different environmental conditions. This study used approved medical devices to measure body temperature and showed that care should be taken regarding how to interpret the results between measurement sites. In addition to contact thermometers, non-contact infrared thermometers could be used to measure temperature [16]. This method of temperature measurement can provide a temperature reading within seconds and it can be performed at a distance from several centimeters to meters away. However, several studies have shown that this method is inaccurate post-exercise [15,35].

In summary, our study emphasized the need for the careful selection and understanding of temperature measurement systems during outdoor exercises. The discrepancies observed between oral, armpit, and tympanic readings highlight the inherent challenges and underscore the importance of continuous evaluation and research in this domain.

## Figures and Tables

**Table 1:** Participants’ information.

	Amount	Average Age (±SD) (years)	Average Weight (±SD) (kg)	Average Height (±SD) (cm)
Male	11	24.90 ± 7.48	74.95 ± 8.40	179.00 ± 7.48
Female	10	25.67 ± 5.85	65.45 ± 13.72	165.50 ± 9.08
Overall	21	25.14 ± 5.80	70.42 ± 11.91	172.57 ± 11.00

**Table 2:** Mean and standard deviation of temperature readings (°C).

Measurement Site	Pre-Exercise (Mean ± SD)	Post-Exercise (Mean ± SD)
Oral	35.83 ± 0.65	35.78 ± 1.41
Armpit	35.21 ± 0.96	35.69 ± 1.58
Tympanic	36.48 ± 0.37	36.78 ± 0.61

## Data Availability

The raw data supporting the conclusions of this article will be made available by the authors on request.

## Correction Statement

Following the removal of this article from the Special Issue “Effect of Physical Activity on Human Fitness”, an Editorial Board member reviewed the entire editorial process and confirmed all elements had been carried out as per MDPI Editorial Process policy (https://www.mdpi.com/editorial_process). As a result, the original Academic Editor listed on this publication has been replaced with that of the Editorial Board member who conducted this review. The original publication has now been updated accordingly. This information has also been transmitted to relevant indexing organizations.
